# Cardiac Development and Transcription Factors: Insulin Signalling, Insulin Resistance, and Intrauterine Nutritional Programming of Cardiovascular Disease

**DOI:** 10.1155/2018/8547976

**Published:** 2018-02-01

**Authors:** Annelene Govindsamy, Strinivasen Naidoo, Marlon E. Cerf

**Affiliations:** ^1^Discipline of Pharmaceutical Sciences, University of KwaZulu-Natal, Durban, South Africa; ^2^Biomedical Research and Innovation Platform, South African Medical Research Council, Cape Town, South Africa

## Abstract

Programming with an insult or stimulus during critical developmental life stages shapes metabolic disease through divergent mechanisms. Cardiovascular disease increasingly contributes to global morbidity and mortality, and the heart as an insulin-sensitive organ may become insulin resistant, which manifests as micro- and/or macrovascular complications due to diabetic complications. Cardiogenesis is a sequential process during which the heart develops into a mature organ and is regulated by several cardiac-specific transcription factors. Disrupted cardiac insulin signalling contributes to cardiac insulin resistance. Intrauterine under- or overnutrition alters offspring cardiac structure and function, notably cardiac hypertrophy, systolic and diastolic dysfunction, and hypertension that precede the onset of cardiovascular disease. Optimal intrauterine nutrition and oxygen saturation are required for normal cardiac development in offspring and the maintenance of their cardiovascular physiology.

## 1. Introduction

Fetal programming and its impact on health and disease is becoming an increasingly prominent area of investigation as studies reveal its close link with offspring health that is shaped by the intrauterine environment of the mother and other key factors. Cardiovascular disease (CVD) encompasses a myriad of diseases that affect the heart and its associated blood vessels, including coronary artery disease that comprises both angina and myocardial infarction [1]. Type 1 diabetes is a polygenic disease triggered by genetic and environmental factors [2]. In type 1 diabetes, an attack on the immune system precedes *β*-cell dysfunction and consequently *β*-cell death [3, 4], resulting in low levels of insulin synthesis and release [1]. Type 2 diabetes is a chronic disease often associated with obesity and sedentary lifestyles [5] and is attributed to insulin resistance in organs resulting in a reduction in glucose uptake [2]. *β*-cell dysfunction also contributes to type 1 diabetes resulting in persistent hyperglycaemia that is exacerbated by insulin resistance. Diabetic cardiomyopathy (DCM) is attributed to altered cardiac morphology and associated with myocardial damage that progresses and leads to heart failure [6] with cell death, a major contributor to DCM [7].

## 2. Cardiac Development and Transcription Factors

During cardiogenesis, that is, embryonic heart development, the prenatal heart grows by hyperplasia, before cardiomyocytes are terminally differentiated after birth, and then grows by hypertrophy [8]. Cardiomyocytes constitute the myocardium and are responsible for contractile heart functions, allowing blood perfusion to multiple tissues and organs [9]. Following cardiogenesis, cardiac growth of the fetus occurs due to proliferation of cardiomyocytes that are mononucleate [10]. In the final trimester and early postnatal life, these cardiomyocytes cease as one or both processes stop, namely, karyokinesis (division of the nucleus) and cytokinesis (division of the cytoplasm) [10]. As a major organ in the developing fetus, the heart is susceptible to cardiac anomalies, which can result in fetal mortality [9] with ∼1% of newborns susceptible to developing congenital heart disease [11]. Thus, during cardiogenesis and early postnatal life, there is an increased risk of developing heart defects and associated cardiomyopathies. Increased cardiac hyperplasia and hypertrophy during fetal development result in an increase in the cardiomyocyte number and size, respectively, thereby enlarging the heart [12].

The four significant steps in murine cardiac development are as follows: (i) the formation of the cardiac crescent (at embryonic day (e) 7.5), (ii) linear heart tube formation (e8.0), (iii) looping of the heart (e8.5–e9.5) and specialized identity of chambers (e10–e12), and (iv) cardiac septation (e12-birth) [11]. The cardiac crescent is represented by a population of epithelial cells developed from cardiac progenitor cells that express cardiac-specific transcription factors [11]. Cells that express cardiac markers merge at the centre of the mammalian embryo [13]. Thereafter, during the early stages of heart development, the linear heart tube forms, which later contracts, enlarges, and initiates several contortions, followed by looping of the heart that shifts the tube right of the embryo and marks the first clear indication of breaking left-right chirality [13]. Thereafter, crucial morphogenetic changes facilitate the formation of the essential chambers, namely, two atria and two ventricles; each chamber is separated by septa, and the atria and ventricles are connected via valves [13].

Transcription refers to a critical event in gene expression and is the intermediary point for the regulation of gene expression [14]. Abnormalities such as altered insulin signalling [15], endoplasmic reticulum (ER) stress [16], mitochondrial dysfunction [17], and inflammation [18] have been implicated in the pathogenesis of diabetes. This may lead to CVD thereby impairing the transcription of key metabolic genes in combination with posttranslational modification of transcription factors that could result in either the activation or suppression of essential target genes [14]. Transcription factors can be categorized into general transcription factors and gene-specific transcription factors; general transcription factors are functional in creating the preinitiation complex which, upon binding to DNA promoter regions, regulates basal transcription [19], whereas the binding of gene-specific transcription factors to genes is sequence-specific and promotes diverse gene expression [20]. Cardiac transcription factor activation is predominantly localized in the myocardium where they regulate the expression of cardiac genes that encode structural or regulatory proteins in cardiomyocytes [21]. The deletion of the key cardiac transcription factor, Forkhead box protein 1 (FoxO1), leads to cell death in embryogenesis due to incomplete vascular development [22]. Forkhead box protein 3 (FoxO3) may positively protect against diseases by preventing both the proliferation and activation of the smooth muscle cells [23], whereas Forkhead box protein 6 (FoxO6) shares some functions with FoxO1 such as stimulating gluconeogenesis that is normally suppressed by insulin signalling [24].

Table 1 summarizes, in chronological order, transcription factors that are essential for cardiac development. The disruption in the expression of cardiac-specific transcription factors during fetal development leads to delayed and compromised cardiac development. Briefly, Hand1 null (at e7.5–9.5) and Tbx5 null (at e9.5–10.5) mice are characterized by arrested cardiac development. The deletion of Mef2C (at e9.5) alters embryonic vascular development thereby inducing vascular anomalies, with CHF1/Hey2 null mice (at e13.5) displaying minor anatomical changes in critical systemic arteries and disordered microvasculature. GATA4 deficient mice (at e10.5) display abnormal ventral folding and inhibition of midline fusion of the primordial heart, whereas FoxO3/FoxO4 null mice (at e10.5–11) have deficient vascular and cardiac growth. FoxO1 is a major transcription factor in cardiac development. FoxO1 null mice (at e9.5) have underdeveloped blood vessels, whereas overexpression of the FoxO1 gene (at e10.5) results in reduced heart size, myocardium thickening (myocardium thickening also results from transgene expression of FoxO1 at e18.5), and eventual heart failure. Like FoxO1, CHF1/Hey2 is another recurring transcription factor during cardiac development. CHF1/Hey2 null mice had thinning of the myocardial wall (at e13.5 and e15.5) with minor alterations (at e15.5) and disordered (at e18.5) microvascular formation. These transcription factors are therefore critical for normal cardiac development and function as compromised cardiac development translates into impaired cardiac function and high susceptibility to CVD.

## 3. Overview of Programming of Cardiovascular Disease

An insult or stimulus during critical growth and developmental periods that disrupts or modifies tissues at both structural and functional levels defines fetal programming [32]. The Barker hypothesis suggests that undernutrition in the fetus, caused by a poor maternal diet (e.g., low protein or hypocaloric diets), which affects nutrient transport to the fetus [33], creates stressors that exert pressure on the fetus to survive, thus forcing the fetus to adapt, restricting its growth and enabling the development of necessary tissues, thereby accelerating maturation [34]. A myriad of environmental stressors such as excess nutrients or hemodynamic forces modify cardiac growth and confer vulnerability on the heart for likely disease later in life [35]. The two critical periods for cardiac development that reflect programming windows are (i) the early embryonic stage and (ii) the late fetal stage when the heart is most vulnerable [35]. The early embryonic stage is highly sensitive to changes in the environment, resulting in modifications in cardiac structure and function, and many heart defects originate during this early stage [35]. Fetal life is a vulnerable stage for increased risk for CVD with potential susceptibility to heart failure due to cardiomyocyte insufficiency and inability, epigenetic alterations, and morphological anomalies [35]. Animal models have been used to study the expression patterns of many genes that contribute to structural defects in the heart [36], although <10% of these underlie congenital cardiac defects in humans [37, 38]. In mammals, in the early embryonic stage when cardiac looping is completed up until the third trimester of gestation, cardiac muscle mass is enhanced predominantly by proliferation [39].

Insults or stimuli affect the health of the mother and subsequently the fetus, and the mechanism by which gene expression is altered at specific sites and tissues in response to these stressors during critical developmental stages is coined epigenetics [40]. Epigenetic mechanisms induce long-term gene expression by alterations in the transcriptional machinery's ability to associate with the chromatin's milieu [40]. Furthermore, these mechanisms do not change the genetic sequence but influence heritable differences or adjustments in the organism's phenotype, and these changes may be transient [41] or durable, that is, persisting long term [42]. There is a distinction between epigenetics and epigenomics: epigenetics is a mechanism that alters gene expression [40], whereas epigenomics is the study of functional elements that regulate cellular gene expression [43]. Considering the definitions of both epigenetics and epigenomics, one can infer the link between environmental insults in the intrauterine milieu and their ability to modify processes at the gene level that can translate into disease phenotypes in the offspring.

## 4. Cardiac Insulin Signalling and Programming

Insulin signalling is an essential physiological process influenced by many internal and external factors. Multiple hormones are implicated in the regulation of fetal growth and developmental programming. Insulin, prolactin, insulin-like growth factor 1 (IGF1), insulin-like growth factor 2 (IGF2), and thyroid-associated hormones are involved in anabolism, whereas glucocorticoids are catabolic hormones [44]. These hormones serve as nutritional or malnutrition markers and function in adapting fetal development to overwhelming conditions in utero, thereby increasing the possibility of survival both in utero and postnatally [44]. The precise physiological outcome depends on the severity, duration, timing, and the type of insult or stressor during development [45, 46]. Normal fetal development can be disrupted by the maternal diet and its associated quality [47]. The excessive expression of IGF2 in mice results in nonspecific organomegaly with abnormalities that include, but are not restricted to, the heart and result in mortality at birth [48].

In the hearts of lean, wild-type mice, glycolysis and glucose oxidation were increased and free fatty acid oxidation was decreased due to insulin action [49]. The insulin receptor (IR) knock-out experiments in mice confirmed insulin's function in cardiomyocytes [50]. Furthermore, a 28% reduction in heart size was observed in mouse models with conditional IR knock-out, which was driven by the creatinine kinase promoter of the muscle [50]. This outcome was due to an analogous decrease in the cardiomyocyte volume [50]. The conditional knock-out of IGF1 and insulin in mice that were driven by the creatinine kinase promoter of the muscle only survived for 3 weeks after birth due to cardiomyopathy and subsequent heart failure [51]. Therefore, IGF1 receptor signalling moderately compensates for cardiac IR signalling during insulin resistance [52].

In a study on lactational programming and insulin signalling, mice were overfed during lactation and displayed increased insulin receptor-*β* (IR-*β*) content, reduced IR-*β* phosphorylation, unaltered insulin receptor substrate 1 (IRS1) content but with decreased phosphorylation, decreased Akt1/protein kinase B (PKB) (Akt1) content, and impaired insulin signalling as there was a decrease in Akt1 phosphorylation as well as a decrease in phosphoinositide 3-kinase-insulin receptor substrate 1 (PI3K-IRS1) interaction [53]. In addition, there was a reduction of insulin sensitivity, elevated cardiac protein tyrosine phosphatase nonreceptor type 1 (Ptpn1-IR*β*) association, decreased Akt1 phosphorylation, and decreased IRS1-PI3K interaction in the overfed mice [53]. Furthermore, in the murine heart, there was an increase of Ptpn1 association in overfed mice that resulted in the impairment of insulin receptor (IR) phosphorylation in the heart [53]. Therefore, the significance of Ptpn1 and its role as a negative regulator of cardiac insulin signalling was confirmed [53]. The development of obesity and insulin resistance in adult mice was shown to occur concomitantly with increased cardiac size and impaired cardiac insulin signalling due to an increase in Ptpn1-IR*β*, a decrease in IRS1 phosphorylation, and reduced PKB-IRS1associated activity [53].

## 5. Cardiac Insulin Resistance and Programming

Cardiometabolic risk encompasses a cluster of risk factors that predispose individuals to type 2 diabetes and premature CVD, associated with disrupted insulin signalling and largely driven by insulin resistance [54]. A reduced response to normal insulin concentrations in insulin-sensitive organs, namely, the liver, muscle, adipose tissue, and the heart, reflects insulin resistance [55]. Insulin resistance can be demonstrated by postreceptor defects at various levels in the insulin signalling pathway [56]. Compared to normal pregnancy, maternal obesity is linked to increased levels of lipid mobilization and ectopic fat in the pancreas, liver, and placenta [57, 58]. Additionally, there is a relationship between increased insulin resistance and obesity during pregnancy [58]. Despite insulin resistance being a great predictor for CVD, it is rarely the sole contributor to the disease [59, 60]. In disease states, such as diabetes and in patients with insulin resistance, the metabolic, structural, and ultimately functional alterations in the heart and vasculature culminate in DCM, chronic artery disease, ischemia, and eventually heart failure [61, 62]. The impairment of insulin-stimulated glucose uptake is the first and steadiest alteration that occurs in the hearts of animal models in the evolution of insulin resistance [63], and this change occurs prior to defects in insulin's capacity to stimulate or elevate Akt signalling, and is attributed to a reduction of glucose transporter 4 (GLUT4) protein in combination with the impairment of GLUT4 membrane translocation [64].

The development of hyperinsulinemia and insulin resistance in murine cardiac hypertrophy is due to pressure overload boosts in myocardial insulin signalling to Akt (in excess), which adds to left ventricular reconstruction at an accelerated level and ultimately, a shift to heart failure [65]. The heart responds to insulin, and insulin resistance is a prominent defect in individuals who suffer from diabetes, obesity, and metabolic syndrome [66, 67].

A high-fat diet (HFD) induced myocardial insulin resistance in C57BL/6 mice within ten days [68]. There was also an association between insulin resistance and decreased glucose uptake in the myocardium, reduced Akt activity, and reduced GLUT4 levels that preceded and was independent of systemic insulin resistance [68]. The consumption of a maternal HFD compromises organ development and renders the offspring prone to metabolic diseases later in life including CVD [69, 70].

In animal models, it was revealed that maternal obesity adversely impacted the offspring, evident by hyperphagia, adiposity, dyslipidemia, hepatic steatosis, insulin resistance, and hypertension [71–73]. In some rat models, a HFD altered breast milk quality as it contained elevated concentrations of cholesterol, protein, triglyceride [74], and leptin levels [75, 76], thus contributing to offspring obesity. Female offspring displayed discrepancies in adiposity, which correlated to HFD exposure in utero and during lactation [77]. Similar to mouse models, rat fetuses maintained on a HFD displayed increased susceptibility to developing metabolic syndrome [78]. Therefore, a HFD contributes to various metabolic syndrome phenotypes characterized by typical metabolic and physiological sequelae induced by insulin resistance. Thus, insulin resistance promotes CVD. A maternal HFD and consequently maternal obesity induces a diabetic phenotype in offspring characterized by adverse effects on fetal heart development and function, thereby triggering offspring susceptibility to CVD that likely manifests later in life.

In myocardial insulin resistance, the rate of fatty acid oxidation remains normal or may be increased, but the rate of glucose oxidation is usually decreased whether insulin-stimulated or noninsulin-stimulated [64]. Reactive oxygen species (ROS) are free radicals and by-products of reduction-oxidation reactions under physiological conditions in eukaryotic cells [79]. An increase in the uptake of lipids and its subsequent oxidation, for example, in insulin resistance, can give rise to cellular lipid intermediate accumulation, excess mitochondrial or peroxisome ROS production, and cardiac derangements, leading to dysfunction [80]. This was demonstrated by the overexpression of cardiac-specific peroxisome proliferator-activated receptor α (PPAR*α*) that induced increased cardiac lipid oxidation and deranged metabolism and subsequently led to both structural and functional alterations detrimental to the heart [81, 82]. The induction of insulin resistance in C57BL/6 mice by maintenance on a HFD also triggered reconstruction of the heart and systolic dysfunction [68]. The heart's ability to tolerate and withstand ischemia and reperfusion can be constrained by myocardial insulin resistance by reducing glucose uptake as well as the synthesis of glycogen and glycolysis, all of which contribute to adenosine triphosphate (ATP) delivery in the ischemic heart for cellular metabolism [83].

Several rodent models mimicking type 2 diabetes and metabolic syndrome display both hyperinsulinemia and insulin resistance in various organs concomitant with cardiac insulin resistance and myocardial contractile dysfunction [49, 84]. In cardiac-specific insulin receptor knock-out (CIRKO) mice, there was a relatively moderate reduction and age-dependent contractile dysfunction [50, 85] that correlated with reduced insulin-stimulated glucose uptake and a reduction in both glucose and fatty acid oxidation as aging and contractile dysfunction occurred [50]. In rodents maintained on chronic HFDs and in insulin-resistant genetic models, for example, ob/ob and db/db mice, insulin had an impaired ability to intracellularly stimulate signalling kinases such as Akt or FoxO1, which in turn caused greater dysfunction of the left ventricle [49]. Insulin resistance alters cardiac adaptation to increasing energy demands causing a shift in the substrate that is utilized as an energy source, with fatty acids being the prominent substrate [86]. In turn, the diabetic heart is subjected to cellular stress, increased production of ROS, mitochondrial dysfunction, and apoptosis with the ultimate outcome of these changes shaped by insulin resistance that then contributes to ensuing structural and functional myocardial alterations, eventually resulting in cardiomyopathy and heart failure [87].

The renin angiotensin-aldosterone system (RAAS), which regulates blood pressure and electrolyte and fluid homeostasis, also plays a role in the pathophysiology of insulin resistance [88]. Consequently, angiotensin 1 (ANG1) is converted to angiotensin 2 (ANG2) through angiotensin converting enzyme (ACE) [88]. ANG2 is the salient peptide of RAAS and its activity has a direct correlation with the pathophysiology of cardiometabolism [89]. Independent of programming, overnutrition stimulates changes in metabolism, thereby altering physiological processes. In the heart, overnutrition concomitant with insulin resistance gives rise to enhanced stimulation of RAAS [90], which consequently supplements elevated activity of nicotinamide adenine dinucleotide phosphate (NADPH) oxidase and an increased production of cytosolic ROS, decreased bioavailable nitric oxide (NO), altered insulin signalling with respect to metabolism, and a dysfunctional diastolic phase [90, 91]. Prolonged overnutrition is a major contributor to insulin resistance in the heart and activates RAAS, uncouples mitochondria, and eventually decreases oxidative stress [92–94]. Obese individuals have reduced insulin sensitivity leading to hyperinsulinemia and eventually dyslipidemia with nontreatment of these conditions increasing obese individuals' susceptibility for developing a diabetic phenotype with an increased risk for CVD [95].

FoxO transcription factors regulate cardiac insulin signalling [96, 97], with alterations in insulin signalling preceding cardiac insulin resistance. In FoxO1-deficient mouse models, embryonic lethality occurs with incomplete embryogenesis, whereas mice lacking either FoxO3 or FoxO4 survive even after parturition [22]. The deletion of both IRS1 and insulin receptor substrate 2 (IRS2) (H-DKO mice: heart-specific IRS1 and IRS2 double gene knock-out) in the brain and liver causes hyperglycemia, but such deficiencies in the pancreas and heart cause organ failure [98]. Thus, there is a high probability that the development of diabetes can cause heart failure due to IRS protein loss [99]. By deleting cardiac-specific IRS1 and IRS2 genes, Akt levels and phosphorylation of FoxO1 are diminished, resulting in organ failure and ultimately death of 7-8-week-old male mice [98]. Heart failure in models overexpressing cardiac FoxO1 mimics heart failure in humans [31]. FoxO1 phosphorylation via PI3K/Akt can be achieved by either insulin or IGF1 [100]. Insulin stimulation prevents gluconeogenesis [101], and Akt represses the transcription of FoxO1 [96]. FoxO1 stimulates and supplements Akt and kinase activity with an increase in Akt by FoxO1, resulting in insulin insensitivity in cardiomyocytes [102]. FoxO1 transcription is regulated by acetylation, phosphorylation, and ubiquitylation [96, 103]. Following the phosphorylation and subsequent activation of Akt by insulin, FoxO1 is phosphorylated and excluded from the nucleus [104]. Furthermore, activated FoxO1 causes metabolic changes altering cell cycle survival [2] through stimulating signalling cascades to prompt cell death [104]. During fasting or low nutrient conditions (e.g., undernutrition), insulin signalling is compromised, leading to nuclear localization of FoxO1, resulting in the expression of enzymes required for gluconeogenesis [104]. During nutrient abundance (e.g., overnutrition), and in insulin-resistant or diabetic states, FoxO1 modulates the oxidation of glucose via pyruvate dehydrogenase kinase 4 (PDK4) [104, 105].

## 6. Programming Stressors of CVD: Intrauterine Undernutrition and Overnutrition

CVD is programmed by multifactorial stressors that influence downstream functions. Fetal programming impacts systemic factors implicated in CVD risk but also has the potential to directly affect the myocardium by mechanical stimulation [106]. There is a likelihood that programming of the cardiovascular system and cardiovascular function in utero is compromised upon a mismatch in growth during the prenatal and postnatal life stages [107]. There are both maternal and paternal influences that program their offspring's health, and in the case of stressors on the heart, the expression of cardiac-specific genes is likely altered reflecting impaired cardiac insulin signalling that contributes to cardiac insulin resistance and the onset of CVD. Normal fetal development and function can be disrupted by a maternal diet; in utero undernutrition and overnutrition represent two common insults for the programming of CVD.

Intrauterine growth restriction (IUGR) is defined as the reduced growth potential of a fetus in utero due an adverse in utero milieu often attributed to reduced substrate supply from the placenta to the fetus [108]. IUGR impacts the metabolic activities of cardiomyocytes and their associated regulation [109, 110], survival [111–113], contractility [114], and cardiomyocyte hypertrophy [111, 115, 116]. Intrauterine undernutrition results in IUGR, leading to low birth weight; IUGR reflects a vascular disorder [117]. Furthermore, intrauterine undernutrition enhances oxidative stress and is associated with impaired endothelium-dependent vasodilation [118]. Poor nutrition (e.g., a low-protein diet) results in restricted growth support and, ultimately, a myocardium with compromised capacity [119]. Offspring of nutrient-limited intake rat dams (dams were fed a 50% ad libitum diet, as established by the quantity of food consumed from the first day of gestation until birth by control rats) develop hypertension concomitant with elevated levels of oxidative stress in the mesenteric arterioles [118].

Chronic hypoxia (over days, weeks, or months) can be induced during early or late gestation through placental embolization [120, 121], placental restriction, and secondary to nutrient restriction [122, 123]. The early fetus is highly sensitive to induced hypoxia, which also causes IUGR, altered gene expression, and cardiac-specific deficits that often lead to fetal death [124]. Maternal hypoxemia (a proxy for hypoxia) can induce fetal hypoxemia that causes IUGR and thinning of the myocardium (due to reduced proliferation) [124]. Male offspring, born to rodent dams exposed to minimal oxygen levels towards the end of gestation were normal at rest but endured critical myocardial damage [126]. Furthermore, hypoxemia-induced bradycardia compromises cardiac output and tissue perfusion, thereby exacerbating hypoxia, with chronic hypoxemia resulting in a thin and disorganized ventricular myocardium, which further compromises cardiac output [124]. These events may feed a downward spiral ending in death due to congestive heart failure [124]. Reduced maternal arterial partial pressure of oxygen (pO_2_) or insufficient oxygen delivery to tissues in the developing fetus results in fetal tissue hypoxemia and hypoxia, thereby triggering changes in fetal development [127, 128]. Hypoxia in utero leads to low cardiac performance and cardiomyopathies that are often present in adulthood [114]. In rodent hearts subjected to prenatal hypoxia, the response to induced ischemia and reperfusion was compromised and characterized by cardiomyocyte hypoplasia but concomitant with cardiomyocyte hypertrophy [126]. Prolonged hypoxemia for the final third of gestation [129] compromised fetal growth and induced smaller hearts with cardiomyocyte hypoplasia [130].

Fetal IUGR, induced by selective ligation of uteroplacental vessels, resulted in cardiomyocyte hypoplasia in both ventricles, hypertrophic remodelling of cardiomyocytes with alterations in microvascularization, left ventricle cardiomyocyte hypertrophy, and diminished capillary numbers and length [8]. These structural findings were associated with fetal systolic and diastolic dysfunction in both ventricles, and upon postnatal challenges such as hypertension, they predispose offspring to CVD [8]. Intrauterine undernutrition enhances oxidative stress and mediates cardiac damage with vascular dysfunction characterized by impaired endothelium-dependent vasodilation and hypertension [118].

**Figure 1:**
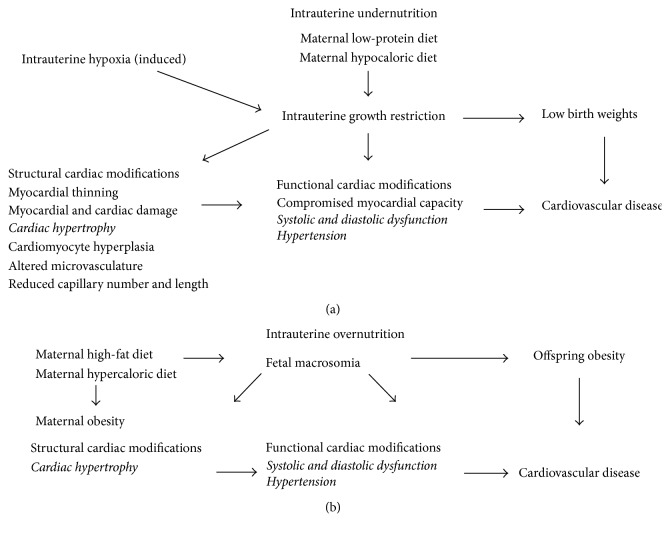
Nutritional programming of cardiovascular disease. Both intrauterine undernutrition (a) and overnutrition (b) alter offspring growth and induce structural and functional cardiac modifications that increase the risk for cardiovascular disease.

In summary, intrauterine undernutrition (Figure 1(a)), such as maintenance on a low-protein or hypocaloric diet in utero, induces IUGR that manifests as low birth weights [131]. A limited supply of substrates restricts fetal growth and delays cardiomyocyte binucleation [115, 119]. Induced intrauterine hypoxia also induces IUGR and consequently low birth weights [131]. Low birth weights are associated with CVD later in life. IUGR results in structural cardiac modifications that include thinning of the myocardium, myocardial and cardiac damage, cardiac hypertrophy, cardiomyocyte hyperplasia, altered microvasculature, and reduced capillary number and length [8]. These structural modifications contribute to functional cardiac alterations such as compromised myocardial capacity, systolic and diastolic dysfunction, and hypertension that often precede the onset of CVD [125].

There is a correlation between maternal obesity and insulin resistance in their neonatal offspring and the future development of certain compromised cardiometabolic states such as offspring obesity, diabetes, and increased cardiovascular risk, demonstrating the detrimental mechanisms of fetal programming [132]. Cardiac hypertrophy is an early consequence of maternal diet-induced obesity that is associated with impaired systolic and diastolic function, impaired ventricular contractility, and reduced myocardial compliance in young-adult offspring of obese dams [133]. In another study, a maternal HFD was demonstrated to further impair diastolic and systolic function in offspring of diabetic pregnancies through lipid droplet accumulation, mitochondrial dysfunction, and oxidative stress [134]. Fetal rats exposed to maternal HFDs (i.e., overnutrition) had increased blood pressure, thereby compromising cardiovascular health later in life [135].

In summary, intrauterine overnutrition (Figure 1(b)), such as maintenance on a high-fat or hypercaloric diet in utero, which contributes to maternal obesity, results in fetal macrosomia and subsequently offspring obesity that increases the risk for CVD. Similar to intrauterine undernutrition, intrauterine overnutrition induces cardiac hypertrophy concomitant with systolic and diastolic dysfunction and hypertension that leads to the onset of CVD. In both intrauterine undernutrition [118] and overnutrition [135], an increase in oxidative stress is implicated in altered cardiac structure and cardiac dysfunction.

Apart from suboptimal intrauterine nutrition, there are several independent metabolic states and stressors that contribute to CVD. Preeclampsia is an independent risk factor for CVD. In addition, gestational diabetes predisposes both the mother and offspring to diabetes later in life, which presents another risk factor for CVD. Metabolic syndrome is a myriad of risk factors that present in individuals who are at an increased risk of developing diabetes or CVD. Maternal smoking during pregnancy altered offspring DNA methylation and mRNA expression, thereby affecting protein expression [136]. The immune response of these offspring was therefore compromised as DNA methylation mediates inflammation and alters leukocyte function, thereby increasing CVD risk [136]. Both maternal and paternal alcohol consumption have adverse effects on the offspring heart. Maternal alcohol consumption confers to offspring a higher risk of ventricular septal [137] and atrial septal [138] defects with alcoholic embryopathy, leading to minor cardiac abnormalities, even without structural congenital cardiac defects [139]. Murine studies have shown that offspring from alcohol-treated fathers have a higher prevalence of low birth weights [140], which would increase offspring susceptibility to CVD. Paternal alcohol consumption may also influence epigenetic impact on the gene expression governing individual organ development [141], and therefore may lead to compromised heart development that could impair cardiovascular function. Paternal alcohol consumption was positively associated with ventricular septal defects in newborn children [95].

## 7. Conclusion

Adverse programming events shape cardiac development, maturation, and function that ultimately lead to CVD. The absence, underexpression, and/or overexpression of key cardiac transcription factors and impaired cardiac insulin signalling contribute to cardiac insulin resistance that often precedes CVD. Further elucidation of the programming of cardiac insulin resistance is required to ultimately prevent, treat, and identify new and/or improved therapeutic targets. Intrauterine nutrition that is balanced and sufficient, concomitant with adequate oxygen supply and delivery to the growing fetus, is critical for normal cardiac development and cardiovascular physiology in offspring, thereby better equipping them to handle stressors that promote the onset of CVD later in life.

## Figures and Tables

**Table 1:** Cardiac-specific transcription factors.

Transcription factor	Embryonic day	Dysfunction/impact/alterations	Reference
(ehand/Hand1/dHAND/HAND)	e7.5–9.5	Embryonic death in Hand1 null mice due to defects in the extraembryonic mesoderm and the yolk sac, followed by cardiac development arrest.	[11]
Mef2C	e9.5	Targeted deletion of Mef2C alters embryonic vasculature development, reduced cardiac endothelial cytokine expression, and resulted in drastic vascular anomalies and embryonic death.	[25, 26]
Tbx5	e9.5–10.5	Tbx5 null mice have unsuccessful looping of the heart and LV hypoplasia, both traits of arrested cardiac development, leading to embryonic death at e10.5.	[27]
GATA4	e10.5	Mice deficient in GATA4 displayed abnormal ventral folding, inhibition of midline fusion of the primordial heart, and endoderm defects with a wide range of lethal effects in embryos.	[28, 29]
FoxO3/FoxO4	e10.5–11	FoxO3^−/−^/FoxO4^−/−^ embryos die due to deficient vascular and cardiac growth.	[22]
CHF1/Hey2	e13.5	CHF1/Hey2 knock-out mice displayed thinning of the myocardial wall with ectopic expression of several genes by the ventricular myocardium that are normally limited to the growing atria and trabeculae on the C57BL/6 background, with only minor anatomical changes in critical systemic arteries.	[30]
CHF1/Hey2	e15.5	Failure in CHF1/Hey2 knock-out mice to enlarge the thin layer of the myocardium and minor alterations in microvasculature formation.	[30]
FoxO1	e18.5	Transgenic expression of FoxO1 led to myocardium thickening, elevated cardiomyocyte proliferation, and reduced p21^cip1^, p27^Kip1^, and p57^Kip2^ expression.	[31]
CHF1/Hey2	e18.5	Erratic and disordered microvasculature in knock-out mice.	[30]

HAND, heart- and neural crest derivatives-expressed protein 1; e, embryonic day; Mef2c, myocyte-specific enhancer factor 2C; FoxO1^−/−^, Forkhead box protein null; Tbx5, T-box transcription factor; LV, left ventricle; CHF/Hey2, cardiovascular helix-loop-helix factor 1/Hairy/enhancer-of-split related with YRPW motif protein 2; PDGFA, platelet-derived growth factor subunit A; PDGFRA, platelet-derived growth factor receptor *α*; *Ang1*, angiotensin 1; PDGFB, platelet-derived growth factor subunit B; PDGFRB, platelet-derived growth factor receptor, beta polypeptide; VEGF, vascular endothelial growth factor; *Ang2*, angiotensin 2.
